# The Hospital. Nursing Section

**Published:** 1904-09-03

**Authors:** 


### The Hospital.

### Nursing Section.

Contributions for this Section of "THE HOSPITAL" should be addressed to the EDITOR, "THE HOSPITAL" NURSING SECTION, 28 & 29 Southampton Street, Strand, London, W.C.

No. 936.—VOL. XXXVI. *SATURDAY, SEPTEMBER* 3, 1904.

### Notes on News from the Nursing World.

### PRIVATE NURSES IN SOUTH AFRICA.

WE draw attention to the serious note of warning which appears in another column respecting the plethora of private nurses in South Africa. Our correspondent is a well-known member of the nursing profession, whose position and knowledge of South Africa give weight to her statements. As she points out, there was a time during the war when it was often impossible to get a private nurse for love or money. Then followed the natural rush to South Africa, with the almost inevitable result that on the cessation of the war the supply greatly exceeded the demand. When a fully-trained nurse accepts £3 a month for salary, which is equivalent to not more than £1 a month at home, there is no need to point a moral to our correspondent's warning.

### A HOME FOR THE NURSES OF SOUTHWARK INFIRMARY.

AT the last meeting of the Southwark Board of Guardians the question of better accommodation for the nurses was raised. The report of the Infirmary Committee, which was read at the meeting, alluded to a statement of the Medical Superintendent that the nurses were not adequately provided for, and recommended that a house near the Infirmary should be rented in order to supply the deficiency. It was observed by one of the guardians, "that at present three or four nurses were crowded into rooms which were only intended for single bedrooms." This, of course, requires to be altered, and it was very properly decided to make immediate temporary arrangements to alleviate the overcrowding. But we are glad to learn that the Infirmary Committee have been asked to consider the question of a permanent remedy in the shape of the erection of a nurses' home. The well-deserved reputation of Southwark Infirmary as a training school is not, therefore, to run the risk of suffering from the reproach that the nursing staff are not properly housed.

### NURSES AND POLITICS.

IT is a matter for regret that at a garden party at Romford, held the other day under the auspices of the Nurses' National Temperance League, the secretary, instead of copying the judicious example of the host, Mr. Alderman Bethell, who did not make a speech, attacked the Government for passing the Licensing Act. There is room for difference of opinion as to the merits and demerits of the Act, but we strongly advise nurses to eschew politics; and if the League is to be worked as a political organisation the sooner it collapses the better. We agree with the president of the Women's Total Abstinence Union, who said that nurses can exert a quiet, restraining influence, and discourage the use of stimulants in the time of sickness and weakness when people are liable to fall into temptation. But this is an entirely different thing from joining in an agitation for the repeal of the Licensing Act. If the Nurses' Total Abstinence League is to be in any sense a power for good, the political element must be rigorously exorcised.

### THE SUPERVISION OF VILLAGE NURSES.

LORD MOUNT EDGCUMBE has very reasonably protested against the view that the useful women employed by the County Nursing Associations should not be designated "village nurses." This is, in fact, their proper title, and so long as the word "village" is used they are not likely to be mistaken for fully trained nurses. The supervision exercised in respect to the County Nursing Associations affiliated to the Queen's Jubilee Institute is, as we showed in our issue of Aug. 6, of a very real character, if the county superintendent discharges her duties in accordance with the regulations. If she does not she can be called to account.

### THE MISSING NURSE AT RIO.

IT will be recollected that several weeks ago we published particulars of the disappearance of a nurse on the staff of the Strangers' Hospital at Rio de Janeiro. The matron of the hospital, writing under date of August 2nd, informs us that nothing further has been heard of the missing lady, and that the Consul-General has taken the case in hand. In the absence of tidings, however, the belief that the unfortunate nurse was accidentally drowned is strengthened.

### THE PROCEEDINGS OF THE SELECT COMMITTEE.

THE official report of the proceedings of the Select Committee on the Registration of Nurses, including the minutes of evidence and appendix, has been issued in a Blue Book, price 1s. The report states that the sub-committee have recommended that a committee be appointed in the next session of Parliament to continue the inquiry.

### THE NIGHTINGALE FUND AND MARYLEBONE INFIRMARY.

THE formal intimation of Mr. H. Bonham-Carter that the Nightingale Fund cannot continue to pay a salary of £10 per annum for 15 probationers in training at the St. Marylebone Infirmary, was fully expected. When the school at the infirmary was founded the importance of adequate training for Poor-law nurses was not recognised as it is to-day, and the Marylebone guardians have rightly appreciated the help given by the Nightingale Fund for all these years. There is now, however, no reason why the whole of the salaries of the nursing staff should not be defrayed, as at other Poor-law infirmaries, out of the rates.

### EXETER GUARDIANS AND DISTRICT NURSING.

The St. Thomas Board of Guardians, Exeter, have recognised the principle of subscribing to district nursing organisations by voting £5 per annum to the Exmouth Association. In the discussion on the question it was pointed out that at Littleham and Withycombe, where the nurses have been working, the cost per head of the population for pauperism is 1s. 4d., while it goes up to 6s. 11d. in some other portions of the St. Thomas Union district. The Rev. F. F. Buckingham, chairman, said that very probably in time contributions of this kind might cost the Board as much as £100 a year, but he thought it would be money well spent. He believed that it was impossible for them to spend £100 a year, or even more, if necessary, in a better way than by seeing that the sick poor were properly nursed. "It was just as much the Board's strict duty to see that the nurses attended the sick poor as to see that a doctor attended them." At Exmouth during the year the nurse attended twenty-four pauper cases and thus saved the ratepayers considerable expense. Upton Pyne and Lympstone now ask for aid and the Guardians have appointed a committee to draw up a scale on which grants shall be made. We hope that the bases will not be on population merely, for it is in the small parishes that it is most difficult to secure private aid and consequently that help is often most urgently needed by the poor.

### NURSING IN PANAMA.

A Scotch nurse, who has gone out to the Canal Hospital, Panama, gives some interesting details of her experiences. The situation of the building itself is on Ancon Hill, several hundreds of feet above the Bay of Panama. The view is magnificent, palm trees abounding, the never-ceasing song of birds and the hum of insects preventing any feeling of loneliness. The staff consists of a lady superintendent and several nurses, each nurse working eight hours a day only, and taking charge of a ward with about 20 coloured patients, most of whom can speak either French or Spanish. The arrangements for the comfort of the nurses are excellent and the food good. The nurses were much impressed by their journey across the Isthmus, which took about two hours, along a single line through thick jungle and hilly country, the foliage being wondrous, with scarlet, purple, yellow, and white flowers in abundance. Bananas grow everywhere, and the little piccanninies running up to the railway cars with their arms full of the golden fruit are strangely picturesque.

### TESTIMONIAL TO THE MATRON OF AN AYRSHIRE HOSPITAL.

Miss M. R. Gay, who for the past eight years has been matron of the Clare Hospital, Largs, has just resigned her appointment in order to go to the United States. Her fine personal qualities and the devoted manner in which she fulfilled her duties, have secured her the esteem of all sorts of people, who took occasion, prior to her departure, to present her with a testimonial as a tangible mark of their regard. Upwards of a hundred persons, representing all classes of the community, subscribed for the purpose, and Miss Gay had the gratification of receiving a valuable gold watch and chain, a dressing bag, and a writing case. The watch bore a suitable inscription.

### THE QUEEN'S NURSE AT ST. NEOT'S.

At the annual meeting of the St. Neot's and District Nursing Association very satisfactory reports were submitted. The district nurse paid 2,821 visits, the number of cases attended being 63. As to the financial position, there was a small balance in hand at the end of the year, and it was mentioned that in several cases the friends of patients have shown their appreciation of the nurse's services by the practical method of increasing their subscription, or giving a donation. The chairman, in the course of a speech in support of the movement, said that nursing associations were "not far behind the British and Foreign Bible Society in doing a great religious work, and people were absolutely of one mind as to their value."

### DISTRICT WORK AT CHESTERFIELD.

At the annual meeting of the Victoria Home for Nurses at Chesterfield the report and accounts for the past 12 months were received and adopted. There is an excellent staff, and they were employed in 407 cases, occupying their time for 439 weeks. The district nurse attended 81 cases, and paid 2,660 visits. We are not surprised that after this she has been ordered by her doctor to take a complete rest. Nor was a sufficient sum of money obtained in subscriptions to cover the cost of the district work. No doubt the collectors find it difficult to make the public understand that the institution is partly a charity. This difficulty is quite usual when private nursing is combined with free nursing of the sick poor.

### DIFFICULTIES AT DEVIZES.

The Devizes Nursing Society cannot be said to have celebrated its coming of age under happy conditions. The twenty-first annual report shows a falling off in the subscriptions, which is all the more serious because it marks a steady decline in the support given to the organisation. In 1900 the subscriptions amounted to £63 16s. 6d.; in 1901 to £60 9s. 6d.; in 1902 to £58 17s. 6d.; in 1903 to £54 11s. 6d.; and in 1904 to £48 4s. As the annual expenditure varies from £66 to £69, and the bank balance, which is gradually getting lower, has had to be drawn upon in order to meet it, the Committee have taken the extreme course of dismissing the nurse, and of going without one during August and September. This is a sad result after 21 years, and suggests that either the collectors are not sufficiently vigorous in their efforts to obtain funds, or that the Devizes people are singularly irresponsive to the claims of an excellent charity.

### SHORT ITEMS.

Miss Flora Giulietta Pauline de Stourdza-Zrinyi has been appointed staff nurse, provisionally, in Queen Alexandra's Imperial Military Nursing Service, and Staff Nurse E. L. C. Crowe resigns her appointment.

### The Nursing Outlook.

"From magnanimity, all fear above;
From nobler recompense, above applause,
Which owes to man's short outlook all its charm."

### AT THE FRONT.

The news that Miss McCane and Miss St. Aubyn return to England next week, and will at once report on Japanese military nursing, is cheering to those who know that, in spite of many advances, our own army nursing still leaves something to be desired. Miss McCane and Miss St. Aubyn have had every advantage, they were allowed to go to the front and see the Red Cross at work on the field, and they also visited Matsyuama, where the Russian prisoners are detained.

As a contrast to their quiet and effective attention to their own business, is the report of the arrival of the American nurses; they were received by the mayor and officials and presented with flowers; they went to a welcome meeting in the church and then to the Grand Hotel, where they had an "elegant" luncheon; then they drove round the city and were greeted with shouts and bows. Next day they went on to Yokohama and were received by the "highest nobility of Japan"; indeed, Miss McGee proudly reports: "Among the company were relatives of the Emperor." They paid official calls and held "quite a conversation" with a Baron, and on one occasion, says Miss McGee, "I talked French to these gentlemen." There were dinners and garden parties in their honour, and a "grand banquet" in prospect when we last heard, but apparently no work—no wounded, no *at the front*—though they had been ashore a week. The only peril of any sort encountered was that Anita Newcomb McGee, President of the Spanish-American War Nurses, was a little sea-sick in her head! This sounds a rather alarming illness, but evidently doesn't interfere with banquets.

Luckily we learn from other sources that the modest and effective little Japanese nurses are doing heroic and devoted service for their country, and are quite capable of looking after their own sick, and are glad to leave the foreigners the entertainments.

Now let us look at what the nurses of the Anglo-American Ambulance had to face at Sedan. There is a story on page 79 of Sir William MacCormac's "Work done under the Red Cross," which shows what went on before the nurses arrived. He says, speaking of a special patient:—"For five days I felt quite sanguine as to his ultimate recovery. Each day he appeared stronger and better. On making my evening visit on the sixth day, however, I found Liprendé weak and exhausted. He said that the attendant had neglected him all day, and that he had had neither food nor wine. It was, unfortunately too true; the rascally *infirmier*, tired of his work, had bolted. The patient died during the course of that night." Further on, on page 86, Sir William writes: "Dr. Sims has already awarded a just tribute of praise to the lady nurses who arrived about this time. I cordially endorse what he has said. I only wish we had had them from the outset; in that case, lives which were lost through want of adequate nursing might have been saved. Women are better adapted, both physically and morally, for the charge of the sick than men. They should, however, in order advantageously to fulfil their mission of good, be adequately trained, and then they become simply invaluable. No male nursing is to be compared to woman's, and I am sure both the French and German soldiers, in our hospital at least, very much preferred it. Female aid cannot, perhaps, be rendered available on the battle-field itself, but there seems to be no reason why it should not be procurable immediately afterwards." The only banquets these nurses received were, as we mentioned a fortnight ago, of dead horse and dirty water! The town was in a state of siege and the work was trying and dangerous, and done under most uncomfortable conditions. But it was done cheerfully and well, and served to show our surgeons the advantage of the woman military nurse.

Then there was the Soudan campaign, in which Sister Emma Durham and Miss Phillippa Hicks and others did such excellent work. Miss Durham devoted herself largely to sick-tent cookery, and was so successful in working cures by diet, not drugs, that in '79 she was sent out to the Zulu war with strict orders to attend to this special department. In the anonymous account of the nurses' work on this occasion we hear of long treks in carts covered in icicles, of furniture made out of packing-cases, and a range consisting of two bricks and an iron bar. Sister Emma nursed in the parsonage at Ladysmith, and Sister Edith in the church. The description of the town may interest some of the nurses who have visited it more recently:—"Ladysmith was a small place, built on the Klip River, almost surrounded by hills. It had one broad street, with the usual South Africa 'sluit,' or wide ditch, running on either side of the street, pretty villa residences surrounded by gardens, separated from the road by a hedge of wild pomegranate and roses." Including the Ulundi wounded, 1,100 men passed through the Ladysmith Hospitals on this occasion and only 29 died. In 1885 there was another Egyptian campaign and Sister Emma, Miss Edwards and others were again at the front, taking things as they came, with such calm remarks as—"I could have slept if the mosquitoes had not been so busy."

And there was the work in the smaller Indian wars and the late South African war; in them all the nurses laboured with quietness and confidence, ever rising higher and higher and becoming more capable, filling their place more assuredly and without any fuss. But it was work, and it was the service of the wounded; and if we have no detailed account of the number of banquets eaten by these nurses, we can only say that the record of cases nursed seems to us more worthy of report.

### Lectures upon the Nursing of Infectious Diseases.

By F. J. WOOLLACOTT, M.A., M.D., B.S.Oxon, D.P.H., Senior Assistant Medical Officer, Park Hospital, Metropolitan Asylums Board, Hither Green.

### LECTURE IV.—SCARLET FEVER.

SCARLET FEVER may be taken as a type of the infectious diseases which are associated with a characteristic skin eruption. There are many such, and as it is part of the nurse's duty to observe and report all such eruptions, a few general hints on the subject are advisable. In the first place, notice the region of the body on which the rash first appears. This varies in the different diseases, so that an accurate account of the early stage may be a useful help in diagnosis. Next observe its method of extension and its distribution about the patient's body. Finally, note its character, its colour, whether it is blotchy or evenly spread, flat to the touch, or raised above the level of the surrounding skin. If it be raised, see whether it consists of solid papules or vesicles. These points, if carefully noted and reported, may be of considerable value, for the rash may alter in character and position before the medical man is able to see it.

The disease known as scarlet fever, or scarlatina, is usually most prevalent in the late autumn and early winter. It chiefly attacks children, and the younger the patient the greater the danger. As a rule it is spread by direct intercourse with the sick, but may also result from contact with infected articles, such as clothing, or from drinking infected milk. The incubation period, *i.e.* the time from exposure to infection to the first symptom of illness is two or three days. It may be a little more or less, but is never more than seven days. The quarantine period, therefore, should not be less than one week. The onset is usually sudden, the first symptoms being headache, shivering, or perhaps convulsions, with sore throat and vomiting. The two latter are very characteristic and are rarely absent. After about 24 hours the rash makes its appearance, at first on the neck and chest and then rapidly spreads to the rest of the body. The colour is scarlet and may vary considerably in depth from a faint blush to a deep "boiled lobster" tint. If examined closely it will be found to consist of two elements. There is a general diffuse redness and thickly dotted over it are a number of tiny points of deeper colour. At this stage, in a well-marked case, the appearance of the patient is very striking. The whole of the trunk and limbs are bright red. The cheeks and perhaps the forehead are flushed, but the parts round the mouth remain pale. The skin feels hot and dry to the touch and the pulse is very rapid. The throat is inflamed and swollen and there may be exudation on the tonsils with enlargement of the glands at the angle of the jaw. A thick white fur covers the tongue, from which the red, swollen papillæ stand out prominently. This is one form of the "strawberry" tongue. As a rule the patient is restless and frequently delirious, and the temperature may be raised to 104° F., or even higher. In favourable cases, after a variable time, improvement begins and rapidly progresses. The temperature falls, the rash fades, headache and pain in the throat subside, and sound sleep comes to relieve restlessness and delirium, so that by the end of a week all the acute symptoms may have disappeared. In other cases recovery is slower or, perhaps, hindered by complications.

The object of treatment in the acute stage is to keep the patient quiet and to make him comfortable. He must, of course, remain entirely in bed, and it is advisable to secure an action of the bowels as soon as possible. He should be encouraged to take plenty of fluid nourishment. Milk with soda-water or barley-water is best, but plain water should not be withheld if asked for. Light solids are frequently given if the patient cares for them. Very often, however, the throat is so painful that all food is taken very unwillingly, and the nurse will have ample opportunities for the exercise of her powers of persuasion. The headache, restlessness, and delirium are best treated by tepid or cold sponging or packing, repeated every few hours. The effect is often satisfactory, good refreshing sleep resulting, while the temperature is kept in check. The throat may be syringed or irrigated with some warm lotion. In the case of adults a gargle may answer the same purpose, and little scraps of ice should from time to time be given and allowed to melt slowly in the mouth. Fomentations or poultices often afford great relief, but it is essential that they should be applied to the right place. The pain being usually felt near the angle of the jaw, it is necessary, in order to obtain the best result, that they should reach round the throat and be carried well up to the ear and fixed by a bandage passing over the head. In this way they completely cover and soothe the seat of pain. If put on in the form of a collar they are all but useless.

By the end of the acute stage, or even before, the rash will usually have disappeared. As a rule, it begins to fade in the parts where it first appeared, and persists latest on the legs. With the fading of the rash, or a little before, the peeling or desquamation period commences. The process starts in the tongue; the fur clearing off from the tip backwards, leaving a perfectly clean, deep red smooth surface from which the enlarged papillæ project. This is the second form of "strawberry" tongue. The skin peels in the same order as the rash developed, the neck and trunk first, then the hands, and finally the feet. Most commonly it will be found that the hands are peeling in the third and fourth week, while the feet may not have finished till the seventh or eighth week or even later. As a general rule, the more intense the rash, the more rapidly will the peeling progress. The process is often very characteristic. The skin becomes dry, and small pinhole-like cracks appear and gradually enlarge into the form of rings, until they join with those adjacent. In this way large areas of skin are rapidly shed. On the feet and hands the skin, owing to its thickness and toughness, will often come away in large pieces. It should be remembered that peeling is not peculiar to scarlet fever, as it may occur after many other rashes, such as erysipelas or measles, for instance. What is peculiar to scarlet fever is the definite sequence in which the peeling commonly occurs and the completeness with which the process is gone through, while the pinhole or ringed form just described is very characteristic.

No special treatment is necessary during the peeling stage in uncomplicated cases. The patient should wear flannel next the skin and should be blanket bathed daily while in bed. His diet must be gradually increased, and in children a good appetite is quickly regained. He is usually allowed to get up at a time varying from 10 days to three weeks from the onset of the disease. In another week or so he is ready for open-air exercise in suitable weather, and should be out as much as possible, care being taken to avoid fatigue and chill. The out-door clothing must be warm, and flannel under-garments should be worn. After getting up he should take a warm bath at least three times a week. The whole process should be gradual, and every step taken cautiously. A minor point may be mentioned. Do not encourage the patient to pick off the thick skin from his hands or elsewhere. Such a practice may result in the

### LECTURES UPON THE NURSING OF INFECTIOUS DISEASES—*Continued.*

production of sores and troublesome cracks. A better plan is to soak the parts in warm water, or to wrap them in lint soaked in glycerine and water. Then rub vigorously with a rough towel or with pumice stone.

So far we have only considered a case of average severity and typical characters. Occasionally the attack is much milder and attended with very little discomfort, and with only a faint rash. The latter indeed may be entirely absent in some rare cases. The desquamation, too, may be very indefinite, especially in young children with soft smooth skins, who are bathed daily. A careful search must then be made for the peeling, and usually the best place to look is the lower part of the abdomen, at the end of the first or during the second week. Characteristic pinholes in the skin are often found in this locality when absent elsewhere. The tips of the fingers at the free end of the nails should also be frequently examined.

On the other hand the attack may be much more severe and attended with great danger to life. Cases of this character may be divided into two groups: (1) *Toxic*; (2) *Septic.*

1. *Toxic.*—The name is intended to imply that the patient has absorbed a large amount of the toxins or poisons of the disease, and, as might be expected, the symptoms are very acute. The outset is sudden, and rapid prostration ensues. The rash is usually very bright and vivid, and the temperature high, while the vomiting and retching continue without cessation. The pulse is quick, and soon becomes weak, and the extremities grow cold and livid. Restlessness and delirium are often present, and coma may follow; the patient, usually a young adult, often succumbing in four or five days, or even less from the beginning of his illness. Such cases are fortunately not common, and the means of treatment at our disposal are rarely of much service. Every effort should be made to economise the patient's strength. He should be kept as quiet as possible, and, of course, not allowed to sit up. To check the vomiting the food must be liquid, and given in small doses. Nutrient enemata are not well retained as a rule, for diarrhœa is a frequent symptom. Stimulants are occasionally useful. The earliest and most obvious sign of danger is the persistence of the vomiting.

2. *Septic.*—This group includes a large number of cases which have all more or less identical features. I will describe a typical and rather severe case. The patient, nearly always a young child, has sharp initial symptoms, with well-marked rash and sore throat. After a few days, instead of improvement, the symptoms become intensified. The early eruption may have faded, and in its place has appeared another rash of blotchy appearance, and most marked on the back, buttocks, elbows, and knees. This is often called the "septic" rash. The throat is much swollen and angry-looking, and the tonsils coated with whitish exudation. Swallowing is intensely painful. The inflammation spreads to the nose and ears, causing pain and purulent discharge. The glands of the neck swell and become tender, and may suppurate. The temperature remains up with frequent fluctuations, and the child is fretful and restless, and rapidly wastes. This state of things may last for days or even weeks, and the patient may die from exhaustion, or as the result of some complication. Recovery depends to a large extent on good nursing. It is often slow and tedious, and the skill and patience of the nurse will be very considerably taxed. The treatment is largely directed to the relief of complications, a part of the subject which will be considered in a future lecture. Otherwise the main indications are to keep up the patient's strength by suitable diet, to check the temperature, and to promote sleep. The nurse who acquires the confidence of her little patients will be the most successful.

### The Venetian Hospital.

BY AN ENGLISH SISTER.

During a recent holiday spent in Italy, we visited the town hospital of Venice. In order to do this it was necessary for us to obtain an introduction to someone well known at the hospital. This we fortunately gained without any difficulty, as we had been introduced to a well-known doctor of the city, who kindly furnished us with the required passport—a letter stating that we were English nurses, and requesting that we might be shown over the building. Arriving as we had been directed, about 10 A.M., we had the morning before us, and the exterior of the building claimed our attention at the outset. The façade is especially rich in ornament, and is considered one of the finest of the early Renaissance school to be seen in Venice. This classical building is generally known as the Scuola di San Marco, having been adapted for the hospital from one of the fine guild schools of which there were so many in Italy in earlier times. It occupies one side of one of the many squares of Venice, and on the right, as one faces the building, stands a fine early Gothic church of the Dominicans, while on the left the water of the canal flows, and gondolas are to be seen in readiness if wanted. When we had sufficiently admired the ancient building—which dates back to the year 1490—from outside, we entered the large portal, where many people were passing in, and we found ourselves in an enormous hall, the greater part of which, however, was enclosed within great gates of open ironwork, where an attendant kept guard. Only a wide passage way was reserved for the incomers, and each one who entered stated his business to an official who sat inside a tiny office to the right of the entrance. Standing aside for a few minutes, before presenting our credentials, we watched the poor Venetians with great interest. Some were evidently out-patients who came armed with the necessary bottle for medicine and ointment jar. Others were visitors for patients, carrying bundles of clothing and often parcels of provisions. One after another passed through, and they were in turn directed into various corridors leading from the great hall.

### Our Guide.

Upon presenting our letter of introduction the official in charge at once obtained a guide for us, and politely told us that we were at liberty to go over the building. Our guide was a young man who wore a blue linen uniform and a cap, with a red badge upon it. He looked uncommonly like a porter, but he proved to be one of the male nurses, these being employed under the religious sisters in all the men's wards. For us the large ironwork gates were opened and we crossed the great hall itself, with its much worn marble floor, wondering why it was not put to some use. It was capable of holding hundreds of people, and has surely been the scene of many mighty gatherings in byegone days.

### Enormous Wards.

When we first entered some of the numerous wards, we were struck by their enormous size and height, and in many cases by the fine decoration of the ceilings, also by the fact that each one contained a large altar. Many of the men's wards contained from 50 to 60 beds, but there was one that contained 93. These occupied what must in olden days have been one of the finest halls of the Scuola. The magnificently carved ceiling and extreme loftiness, must make it unique as a ward. Beds were arranged on both sides and at one end of this great hall, and then two rows of beds were placed head to head down the centre. At the other end was an altar quite as large as one would see in a very large Roman Catholic Church. The windows, though very large, were higher than tall people could see anything out of, except the cloudless sky, and standing as we did at the foot of the altar overlooking the four rows of beds, every one of which was occupied, and one's line of vision unbroken by any subdivision of the ward, by screens or any colour, one could not help feeling depressed. I have never felt a hospital ward so sad a place at home as I did in "sunny Italy." It may be that the contrast between the brilliant sunshine, the picturesque colouring, and treasures of art that are so attractive outside are not the best preparation for a glimpse of a "palace of pain."

### Children with Shaved Heads.

The women's wards were smaller, and the children's wards contained about twenty cots. These wards were also inexpressibly sad. So still and quiet; and except that in the case of the very sick children the mothers sat beside them, there was no attempt at any amusement for the little ones. Yet there was evidence of good care being taken of the children. The beds were white and clean, dressings in the surgical ward were beautifully put on, and cleanliness reigned apparently everywhere. Every child in bed had its head shaved, and this no doubt added to the general effect of the extremity of illness. Such treatment would make a number of healthy children look rather ill. Unfortunately, it is a rule that has to be enforced among the little patients. It was with some relief we turned to the convalescent ward for the little ones, where several pretty children were happily playing. This room had long low tables and seats adapted for the use of the small people at meal times, arranged round the sides of the room, leaving plenty of space for a good romp in the centre.

### Cleanly but Bare.

All the wards, and indeed all the building, had flooring of mosaic—the general appearance being one of great cleanliness. Beside each adult bed stood a bed-table with a wide shelf underneath—table-top and shelf both being of glass. The table itself held the patient's allowance of milk, in a bottle, with cup or feeder, etc., and on the shelf underneath, not always neatly folded, were the patient's clothes. Utensils were to be seen under the beds, so that although the floors were well washed, and the beds with their white covers looked clean enough, the wards entirely lacked the trim appearance we so much pride ourselves upon in England. The altars which were to be seen in every ward were without exception very beautifully kept, their care probably being a labour of love on the part of the religious sisters who are in charge of the wards. Quantities of artificial flowers are used to decorate these altars, natural flowers being scarce and ruinously expensive in Venice.

### The Sisters and Nurses.

The sisters wear a simple black uniform with large white apron and sleeves, and the community veil over the closely-fitting white cap. They were always very busy, and smiled and bowed to us as we passed into the wards, but did not attempt to come and speak to us or to show us round. The female nurses who work in the women's and children's wards belong to a very poor class; they are paid by the day, provide their own food, and do not sleep in the hospital. In one ward, where the morning work was evidently just finished, we saw two of these nurses sitting on a table at the end of the ward, eating food from a basin with their fingers. In another ward we noticed one of them bandaging a patient's leg beautifully. All the bandaging we saw was very well done, and in almost every case washed bandages were used. These nurses wore cotton blouses and skirts, with large white aprons and sleeves, and a white kerchief pinned across the shoulders, forming a kind of white cape. They did not wear caps, but the Italian women mostly have pretty hair, with a natural wave in it, and many of them looked picturesque enough, especially as the cotton gowns, though made in the same style were of various colours. The male nurses in the men's wards all wore the blue linen uniform, such as our guide was wearing.

### The Operating Theatres.

After leaving the wards we were conducted through a very long corridor, across a great courtyard, into a fine church, belonging to the hospital, which was typical of Italian churches, full of pictures and monuments of marble. Then we saw the dispensary, and afterwards two small operating theatres. One of the latter had just been used, and as the guide threw open the doors the scene was somewhat gruesome in the bright sunlight. The other, however was in perfect order, and the man pointed out with intelligent knowledge the precautions taken to ensure aseptic treatment. All the furniture was of the simplest description possible for its purpose, but everything was washable or able to be sterilised—the dressings were in sealed boxes, lotions (coloured) in glass jars. Taps could be turned off with the elbows, and nail brushes stood in glass dishes. The room, like the wards, was very lofty, windows far too high for us to look out of. But when we had examined the details of equipment, one of us remarked upon the excellent light, as we looked upon the intensely blue sky seen through the large windows. Our guide smiled, and taking the steps from beside the operating table, he asked us to mount them, and look out of the windows, and he stood waiting in evident anticipation of our praise. And no wonder, for a scene of perfect beauty delighted us indeed. Glittering in the glorious sunshine we saw a broad stretch of water, this side of the building bordering on the lagoon, and in the distance we could see parts of the "Island City," its marvellously picturesque colouring made especially beautiful because of the perfect day.

### The Laundry and Kitchen.

From this beautiful scene we were taken to the prosaic region of the laundry. Two very sweet-looking sisters were in charge, they sat writing at a table, busily watching at the same time the men who worked the machinery and women who were doing hand work, for their little recess was so placed that they could see almost all that was going on. The men wore the blue linen uniform, and the women the same kind of dress as those who worked in the wards. Finally, we visited the kitchen and store-rooms. In this department sister came forward and conducted us round herself, the man falling behind in quite a reverential manner meanwhile. The kitchen was very large, and several women were cooking. The entire centre of the room was occupied by the stoves, which are quite different from those we use. So also is the cooking, as, when possible, the national dishes are allowed. All the utensils in the kitchen were most beautifully kept; large copper cans, polished to perfection, stood in readiness for the soup, etc., which was almost ready to be served, while for the wards near at hand trays with the requisite number of basins were prepared.

### Store-rooms and Larders.

In the store-rooms and larders, which sister showed us with pardonable pride, we saw many things that proved the difference in the feeding of patients. Bread, for instance was made in the little closely kneaded, and somewhat hard-baked rolls such as is eaten by the poorer Italians. A loaf from which one could cut an ordinary slice of bread and butter was not to be seen. But there was a large machine for grating bread, and judging by the quantity prepared, much of the bread is used in this form. Another machine was for grating cheese, of which large quantities were also ready. Macaroni of every variety we also saw, and grain of various kinds. Some vegetables, of which we did not know the names, and fresh salad were being prepared in one of the rooms. We could have watched the proceedings with pleasure for a long time, but the morning was already passed, and we could tell the dinner hour was approaching. So we took leave, much charmed by sister's gentle courtesy, She seemed so happy in her department, and the quiet careful way in which her subordinates worked, gave evidence of her skilful management.

### Nursing in South Africa.

### A NOTE OF WARNING.

It is just over a year now since my return to South Africa to take up private nursing again, after a year in England to recruit from the effects of the campaign. During this year, in the nursing world, things have gone gradually from bad to worse. I am, and for five years have been, a member of a well-known institute in one of the largest towns in South Africa, and noticed, immediately on my return, that the number of nurses waiting for work in the institute, was greatly in excess of what I had ever seen before the war. For a short time, in the enteric season, matters apparently improved so much that more nurses were encouraged by the authorities to come out and swell the number of the insufficiently employed, with the result that in this town there is at present a still worse slump in the nursing market than a year ago. The slump is also very serious in Johannesburg, Pretoria, and more or less in South Africa generally. When I left this institute for a case three weeks ago, there were 11 or 12 nurses on the list for work, and when I returned a fortnight later the list had increased to 15, some being still on the list who were on it when I left. During the three weeks I was away, at one time the list of unemployed numbered 18, this out of a total of something like half a hundred. There are other nursing institutions in the town, and also a great number of private nurses working on their own account.

It is perhaps difficult for people in England to understand that such can be the case, especially if they are encouraged by those in authority to come out; but, for this, reasons may not be far to seek. It pays the heads of institutes to have a large number of nurses on their books, for when the nurses are employed a high percentage accrues to the institution, and when unemployed nurses pay highly for board and lodging in the institute to which they belong. It might be thought that such a course of reasoning applies only to such institutes as are run by private individuals for their own profit, but this is not always the case. This is, or was, a money-making country, and the committee or head of an institution, originally started ostensibly for the benefit of the public and nurses, may become fascinated by the idea of making money, the benefit of the nurses having become a secondary consideration. During the war it was often impossible to get a private nurse for love or money, and then many came out to take the place of those who had gone to the front. Now a great many of those working for the military have been discharged, and of these many who came out from home or who came from other colonies have remained in this country, in addition to those originally here. As a consequence the market is overstocked, and likely to be more so. The new military system is not yet in full working order, and nurses still employed in that service will later also get their discharges. In October next the staff of military nurses is to be reduced. South Africa is no longer an El Dorado for nurses, some, indeed, can hardly earn a living, and I know of a case where a fully-trained nurse in this town is accepting £3 a month for salary. How many more of such cases there may be I cannot say.

### Everybody's Opinion.

### A TRIP WITH A FEVER AMBULANCE.

"A Fever Nurse" writes: My experience with a fever ambulance may interest your readers. Suddenly the maid called out "Nurse, ambulance!" I hurried to dry my hands, rushed to my room, put on my long blue ambulance gown, took my basket of stimulant and blankets, jumped up, and was away in less than five minutes, the time allowed. We arrived at our destination in the East End of London, where, to my surprise, there were quite 100 curious mothers with their children collected round a dark court. I stepped out. I was welcomed with the remark, "Here she comes! ain't she clean?" My driver whispered to me to be careful, and said, "They are a funny lot in this court; the last room on the top floor." I climbed up to it and found Tommy, my patient, fully dressed. I took off all his clothes and wrapped him in the flannel gown and blanket. After much weeping, his mother implored me to take care of him and be kind to him. Then I discovered that in my hurry to get Tommy in the blanket I had allowed him to retain a slice of fat, raw bacon round his neck, this being regarded by his parents as a sure cure for sore throat. I took it off, to their disgust, and we reached the door. Here three halfpence were thrust in my hand, with the explanation, "To get you half-pint, nurse!" I gave it back with many sweet smiles, and explained that we were not allowed to take presents of any kind. Outside, my ambulance was besieged with curious admirers, though a few mothers told their children to stand clear. After a struggle through the throng, who held their noses, some with dirty handberchiefs and some without, I got inside with my burden. My driver shouted to the admiring crowd to make way, slammed the door, and at last we drove away to the hospital.

### Appointments.

London Fever Hospital, Islington, N.—Miss Emma S. Harrison has been appointed night sister. She was trained at the North-West London Hospital. She has since done some private nursing and has been holiday night sister at the Hospital for Diseases of the Heart, Soho Square, London.

Royal Hospital for Diseases of Chest, London.—Miss Lilian Lloyd has been appointed night superintendent. She was trained at the Jessop Hospital, Sheffield, and the General Infirmary, Bolton, Lancashire. She has since been charge nurse at the Park Hospital, Hither Green, London, and night superintendent at the Royal Infirmary, Halifax.

St. Mark's Hospital for Fistula, City Road, London.—Miss Clare E. Brunt has been appointed night sister. She was trained at the County Hospital, Newport, Mon., and has since been night superintendent in the same institution. She also acted as district nursing sister with the West London Mission.

### Sketches of Our Hospital:—A Medical Ward.

BY E. MARGARET FOX.

It lacks the brightness of a surgical ward, for here, the patients are, as they themselves express it, "more bodily ill."

There is a chastened air about it, and on some of the faces a look of grievous illness. The bedclothes are not disturbed by lofty erections known as "cradles," but here and there is a multiplicity of pillows, against which lie, propped up, blue-lipped, anxious-looking faces, wrestling hopelessly, with irregular gasps in the struggle against the great enemy under one of his dread guises of heart disease.

No less terrible, but more insidious, is he in his method of attack on the prostrate occupant of that bed, who, with twitching fingers, is picking aimlessly at the quilt, and whose dull eyes, dry lips, and low, unmeaning mutterings, point to the last stage of typhoid fever. His neighbour, a lad of fifteen, with the sunburnt face and healthy look born of much time spent in the hospital garden, is the "chronic" and the oldest inhabitant of the ward.

Admitted, in the first instance, under a physician, his case proved, to speak professionally, to be "medical, requiring surgical interference," and as such he is most impartially bandied from one ward to another, according to the pressure on the beds, frequently sleeping cheerfully on the couch, when the wards are so full that there is no bed at all for him.

He is always "soon going out," but somehow he does not go, and has come to be regarded almost as a part of our hospital. It is he who instructs new patients in the mysteries of ward routine, and he is the trusted ally of every fresh probationer, who appeals to him invariably as the exponent of her duties, if she happens to be too shy to ask the ward sister. A new night nurse finds him invaluable, for he always knows where everything is kept, and which patients are able to wash themselves, besides other priceless bits of information to the new-comer.

There are two or three cases of rheumatic fever in this ward—fine, strong-looking young fellows, but reduced to the helplessness of babes with illness, their pain-racked limbs swathed in wool and bandages, and jarred by even the gentlest movement.

Coughing sharply and ineffectually is a case of pneumonia. He has not yet reached the crisis, and his temperature is high, and his mind clouded with delirium. His bed is protected by padded boards at the sides to frustrate his incessant attempts to escape, and between each sharp cough issues a petition for someone to "bring his clothes."

There is a screen at the foot of the next bed, and its occupant is very quiet. The outline of his emaciated form is sharply defined under the white quilt, and by his side, an infant in her arms, sits his wife, mechanically rocking it to and fro, but with eyes for nothing except that face with the unmistakable Shadow gathering upon it. He has been a long time in the ward, an originally iron constitution contesting every inch of the ground now so nearly won from him, and to the last he is battling for his life with bent brows and clenched fingers, though the hopelessness of defeat now looks out from his sunken eyes.

All unconscious of the impending event next him, a bright young lad, who is rapidly regaining the bounding pulse of healthy life after a severe illness, plays draughts noisily with a thin-faced convalescent typhoid, the progress of the game being closely watched meanwhile by two more patients on the big couch opposite. Some nurses come round presently, and somewhat unceremoniously displace the draught-board.

"Oh, Jimmie, how untidy your bed is, to be sure! Come, lie down, now, and let us put it straight, and then you must keep still, because your doctor will be here very soon."

Jimmie gives way with a sigh. He does not like Monday afternoons, for then he has to be very quiet for a long time, while the physician makes a lengthy round, and nurse will not even let him have *Comic Cuts* to look at, ever since he rustled its pages unduly, one day during the doctor's examination of someone's chest, so that the "breath-sounds" could not be heard properly. He watches the beds being carefully tidied, with sundry clean sheets added here and there, and sees with regret how all the magazines are ruthlessly gathered up and put into lockers out of sight.

Tea is just over, and to-day, none are allowed to keep their mugs for an indefinite time. An inexorable probationer, deaf to all entreaties, compels their immediate return to the tray she is carrying round, and flatly refuses a third replenishment to anyone. The convalescent typhoid, still keenly hungry after his meagre allowance of regulation "bread and butter without crust," gazes disconsolately at the vanishing plates, and longs to repeat Oliver Twist's request for "more," but for the certain assurance from past experience that it would be of no use.

The physician's step is heard in the corridor, some ten minutes or so before his time, and the ward sister looks worried because everything is not in perfect order. She pulls a stray quilt straight as she passes and tucks an offending red handkerchief out of sight under a pillow. The probationer motions one or two convalescents to their respective chairs by their beds, and they respond with laboured efforts to tread quietly, which result in a good deal of noisy scuffling. Then there is silence in the ward, except for the delirious mutterings of the man with pneumonia, and an irrepressible cough from a patient now and then. All eyes are on the little group passing slowly from bed to bed, and all ears are eagerly listening to what the doctors say. But they talk together in low tones, and only now and again are their words audible to the occupant of the next bed.

"Let me see the chest, please, nurse," and up and down the stethoscope moves, while the patient is commanded to "say ninety-nine—again—now, count one, two, three," and so on, through the process described by one patient, who said that "the doctor played the piano on his chest, and listened to music through some tubes"! The stethoscope passes from one hand to another, and much talk ensues of "râles," "murmurs," "systolic bruits," and such-like. Jimmie gets restive, and begins a whispered conversation with his next-door neighbour, only to be frowned down immediately by the sister. The pneumonia's requests for his "clothes" are so urgent when the group reaches his bed, that it is with difficulty he is restrained from leaping out in search of them. It is of no use to tell him to say "ninety-nine." He says instead, incessantly, "Gimme my clothes," and the physician has to auscultate upon that. A message is brought to the ward presently, of "an urgent case—man of forty—can the resident take him in"? He looks round at all the beds, occupied with cases that cannot be sent out yet, safely, with something like despair. Then his glance falls upon Jimmie. "He can sleep on the couch," he says to the sister, adding to the messenger, "I'll take him. Bring him along." Jimmie, hugely delighted at the change, is bundled on to the already full couch by the probationer, while his bed is hastily prepared for the new case, who arrives on the stretcher before the physician's round is finished. His face is grey, pinched, and anxious. It does not need more than a glance from the experienced eye to see that his case is indeed "urgent," and the little group quickly closes round his bed, while someone goes to fetch the surgeon.

"Theatre?" whispers the sister to the resident, in an aside. He nods, and straightway the probationer goes noiselessly out of the ward to prepare for the operation that must soon take place. The surgeon for the week, who was in the next ward, comes in presently, and with the deft and supple fingers of a most flexible hand, makes his examination in silence. He and the physician consult together briefly afterwards—very briefly, for their decision is unanimous—"Operation at once."

"How soon can you have him ready, sister?" asks the resident, and receiving the prompt reply, "In ten minutes," the examination of the patients is continued, while two nurses disappear behind the screens, and the little group moves towards the door. They stand there talking together for a few minutes, while the sister, at a sign from the pallid-faced woman, sitting by that other screened bed, goes to her side, and looks at the drawn, white face on the pillow, so grave and still in its moment of defeat.

She comes back to the group at the door, a reflected gravity in her own expression, and waits her opportunity to speak.

"Who is likely to be going out, soon?" the physician is asking the resident. "I have a case that ought to come in, but there doesn't seem much chance of a bed at present." The sister indicates the screen with a look, and he following her glance, understands.

"There is an empty bed now, sir," she says quietly.

### The Nurses' Bookshelf.

Lost Angel of a Ruined Paradise: A Drama of Modern Life. By the Very Rev. P. A. Sheehan, D.D., Author of "Luke Delmage," etc. (Longmans, Green and Co. 3s. 6d.)

Dr. Sheehan has written his drama for the benefit of the sick children in the Temple Street Hospital, Dublin, and all proceeds will be devoted to the new convalescent home in connection with it. The motive of the drama is the working out of the careers of three Irish schoolgirls, who appear in the first chapter as the Parcœ, or Fates (Clotho, Lachesis and Atropos), in a Greek play which is being played at the close of their last term at school. Grace O'Meara, who as Clotho holds the distaff, marries Dr. Latouche, a London practitioner; Eva Farrell, Lachesis, finds her vocation in convent life, and Lilian White, Atropos, after some vicissitudes, and having been painted by a Parisian artist, inspired by a chance meeting to make her the model for his picture, "Lost Angel of a Ruined Paradise," becomes a hospital nurse. The drama is a curious medley in which intrigue, jealousy, love and religion, are mixed in equal proportions. The book is particularly well got-up, and the emerald green cover makes an attractive and suggestive exterior.

The Holidays, 1904; Where to Stay and What to See. (London: Walter Hill.)

The enterprising publisher whose name appears on this useful book, has conceived the excellent idea of amalgamating all the railway holiday *brochures*, so that holiday-seekers can study at their ease all the different guides belonging to our main railway lines. Hitherto we have been obliged to collect around us six or seven of these guides to "seaside, farmhouse, country lodgings, and hotels," now we can furnish ourselves with Mr. Hill's "Where to Stay and What to See," and feel fully equipped. The illustrations, as in former years, are good and the information furnished in the descriptive part extremely useful and gives what we need, but the fault of the book is one in no way attributable to the compiler. In hardly one case is there any indication given as to the terms asked by the various candidates for holiday visitors. This is entirely the fault of the lodging-house and hotel-keepers. They resist to the last drop of their blood giving any idea of what their demands on our purses will be. It is a signal mistake, and experience shows that we should often take a short holiday in the country if we knew to within a few shillings what our expenses would be. As it is, we have perhaps an unexpected three days to call our own, but as two days must pass ere we know if our would-be landlord's terms will suit our purses, time flies and we stay at home. If landlords would frankly say what they ask, they would soon see their circle of visitors enlarge. The Great Eastern Railway certainly stands first in its practical way of telling us as much as possible at a glance. Not only does one see the price of the railway ticket, return fares to every place mentioned, but also the length of time which all the different tickets allow us at our chosen resort. This is a great advantage, and the more slender the purse the more useful such information becomes.

### Death in Our Ranks.

We regret to record the death of Miss Mary Wade, superintendent of the Hamilton Branch of the Q.V.J.N., which took place on August 25th after a very short illness. Miss Wade entered the Royal Infirmary, Edinburgh, in April 1890, and after her training joined the Queen's Nurses in the District Home in Edinburgh in September 1893. In March 1894 she was appointed Queen's Nurse at Peebles, where she worked till September 1899, when she was transferred to the post which she held until her death. Her whole nursing career was characterised by an intense love of her work and of her patients, and a shrinking from any publicity or praise, which makes it difficult to write what one would like to say about her. Both at Peebles and in Hamilton she was deservedly beloved by her patients and respected by all. She was only ill for 10 days, and died of an illness the result of a chill caught in the discharge of her duty.

### TRAVEL NOTES AND QUERIES.

By our Travel Correspondent.

The St. Louis Exhibition (L. W.).—I fear that there is no possibility of doing the journey without heavy expense. Write to Messrs. Cook, Ludgate Circus, and ask for all their papers relating to the Exhibition and also their "Ocean Sailing List." Look particularly at page 25. There are conducted parties being taken; cost from 30 guineas. As travelling in America is very expensive, your young friends would do well to avail themselves of one of these parties in my opinion. Hotel charges are such as to give the possessor of a slender purse the nightmare. Certainly they could go safely steerage and it is quite clean and respectable, but I do not advise it. Stamps returned.

Rules in Regard to Correspondence for this Section.—All questioners must use a pseudonym for publication, but the communication must also bear the writer's own name and address as well, which will be regarded as confidential. All such communications to be addressed "Travel Editor, 'Nursing Section of *The Hospital*,' 28 Southampton Street, Strand." No charge will be made for inserting and answering questions in the inquiry column, and all will be answered in rotation as space permits. If an answer by letter is required, a stamped and addressed envelope must be enclosed, together with 2s. 6d., which fee will be devoted to the objects of "The Hospital" Convalescent Fund. Any inquiries reaching the office after Monday cannot be answered in "The Hospital" of the current week.

### Echoes from the Outside World.

### Movements of Royalty.

The King is said by Dr. Ott, his Majesty's physician at Marienbad, to be enjoying the best of health. "Appetite, sleep, and digestion," Dr. Ott states, "are excellent, his spirits high, and he feels stronger than ever." On Friday the King witnessed the last performance of the Bayreuth artists in the parts of "Lohengrin" and "Die Meistersinger"; on Saturday he went for a long automobile ride, dining in the evening at the Kursaal Restaurant. On Sunday morning he attended service at the English church, and in the evening entertained Prince Louis of Battenberg, the British Ambassador at Vienna, and others at dinner. On Monday his Majesty devoted the greater part of the day to the transaction of State business, omitting even his usual motor drive. He is due in London on Saturday morning. The Queen has been spending her time very quietly at Mar Lodge, enjoying a thorough rest in the Highlands.

### The Tsarevitch.

On Saturday an Imperial decree was published directing that, in commemoration of the birth of the Tsarevitch, a sum of money shall be assigned from the Imperial Privy Purse sufficient to found 100 scholarships, to be divided equally between naval and military educational establishments, and to be named after the Emperor and Empress, for the children of deserving soldiers and sailors wounded or killed in the war. The administration of the Imperial estates is also directed to set aside the annual interest on a sum of 2,000,000 roubles from the funds under its control, in the name of the whole Imperial family, for the support of the families of deserving soldiers and sailors, to be used preferably for the education of their children.

### The War in the Far East.

Reports from General Kuropatkin and General Sakharoff as to the operations of Thursday and Friday last week indicate that the Japanese were pressing heavily on the Russian southern and eastern fronts, but that the attacks were for the most part repulsed. To the eastward, however, General Sakharoff states the battle became more serious, involving a loss to the Russians of 1,500 men and six guns. There was fighting throughout the whole of Friday outside An-shan-chan, and the Russians were driven from their first line of defences. They lost in their retreat a major-general, and a lieutenant-colonel, and the troops waded through a sea of mud amid a blinding rainstorm.

### The Emperor and Crown Prince of Korea.

The Emperor of Korea, the Hermit Kingdom, who has just received the foreign correspondents of some of the English papers, is described as a short, somewhat stout man, of about sixty, with black hair. He wears the typical Korean moustache, curling downwards, and meeting the hair on his chin at the corners of his mouth. His smile is said to be very pleasant and exceedingly affable. The Crown Prince appears to be very different to his father, to be much fuller in the face, and without the Emperor's charm of manner. He did not take any notice of the obeisances made to him, moved uneasily from side to side, and displayed, it is stated, an entire lack of dignity pitiful to behold. Moreover, some natural mistakes made by people who bowed to the chief eunuch of the Palace, who was the only person on the daïs besides the Emperor and his son, while causing amusement to the former, evidently gave considerable annoyance to the Crown Prince.

### Premier's Prize to British and Dutch Children.

The Loyal Women's Guild of South Africa has received, through the Victoria League, the offer of a prize by Mr. Balfour, to be competed for by British and Dutch children, for the best essay on the British Empire, with special reference to the following points:—What is the ideal of Government represented by the British Empire? How does the British Empire differ from the empires of the past? What should be the relationship between a British colony and the mother country? What should be the attitude of the citizen of a colony towards the empire? How can the unity of the empire be best promoted and maintained?

### Death of Dean Hole.

The death of Dr. Samuel Reynolds Hole, Dean of Rochester, took place early on Saturday morning. The dean, who was 85 years old, had been declining in health for some time, but after a relapse at Easter, he rallied so much that he was able to receive visitors. He preached his last sermon in the cathedral on Christmas Day. It is no reflection on living deans to say that the venerable ecclesiastic who has just passed away was the most widely beloved of his order, and was regarded with esteem by all sorts and conditions of men. In the words of the late Archbishop Temple, he was "the very best type of dean—a literary man, a portly man, a popular man, a good rider, an excellent preacher, a born humourist, while he was the greatest admirer and best judge of the sweetest of flowers." Even his ability as a preacher, and his reputation as a master of sarcasm, were not greater than his fame as a rose-grower, and his "Book about Roses" is known and read everywhere. The genial author was visiting one of the most beautiful gardens in England during the absence of the owner. Meeting the head gardener, he asked permission to make an inspection of the place. "Are you Mr. Reynolds Hole," inquired the gardener. The dean, having replied in the affirmative, was somewhat perplexed to see the man turn his back. His perplexity, however, was quickly relieved on hearing the gardener shout out to one of his assistants, "Set the fountains playing, Bill."

### Postage-Stamp Exhibition.

There was opened at Berlin last week an International Stamp Exhibition, which is naturally of great interest to collectors. The collection which attracted the most attention on the opening day was that of Mr. H. J. Duveen, of London. It embraces the very rare Mauritian stamps, the penny red and the twopenny deep blue, which together are valued at £2,500. The British Guiana set in the collection contains the rare pink 1840 two cents. The British exhibitors are very numerous, and include Baron de Worms, whose collection of Ceylon stamps is noteworthy; Bright and Son, London; Messrs. Beckton, Manchester; Mr. Haymann, West Hampstead; Mr. Wilson, Birmingham; Mrs. Frances M. Bridson, Dartmouth; and Mr. L. L. R. Hausburg, of Weybridge, whose collection contains a fine representation of South Australian, West Australian, and Indian stamps.

### New Play by Mr. H. A. Jones.

On Saturday evening a play, described as a new and original comedy, by Mr. Henry Arthur Jones, was produced at the Garrick Theatre. It is called "The Chevalier," and partakes much more of the character of a farce than that of a comedy. The interest centres in the Chevalier Monteagle, represented by Mr. Arthur Bourchier, who is rarely absent from the stage and fills a very difficult part with great skill. The slender plot is of quite minor importance, and but for the liveliness which Mr. Bourchier displays from first to last, the play would not stand the slightest chance of remaining on the bill for any length of time. There are three acts, and noisy nonsense is the predominating element of them all.

### Notes and Queries.

**FOR REGULATIONS SEE PAGE 143.**

*Abroad.*

(167) Would you give me the address of nursing homes at St. Remo, Cannes, Mentone, Nice, and Cairo? I am anxious to get work abroad during the coming season.—*Salome.*

The English Nurses' Institute and Medical Home, Sunnybank, Via Borgo Pescio, San Remo; the Nice Nursing Institute, Avenue Désambrois, Nice, etc. See Burdett's "The Nursing Profession" for others.

*Board-Residence.*

(168) Would you kindly tell me where a respectable young woman could have board and lodging whilst seeking work in London? A home such as is sometimes connected with the Young Women's Christian Association or Girls' Friendly Society would be suitable.—*A. G. P.*

Write to the Secretary of the Girls' Friendly Society, 39 Victoria Street, S.W., or to the Secretary of the Young Women's Christian Association, 26 George Street, Hanover Square, W.

*Electrolysis.*

(169) Can you kindly tell me if electrolysis effectually removes the hair from the face, or does it encourage a fresh growth?—*L. M. F.*

Electrolysis properly performed permanently removes the hair.

*Hair Specialist.*

(170) I should be grateful for the name of a London hair specialist. Mine has suddenly gone grey.—*L. M. F.*

Let your doctor advise you.

*Home.*

(171) Can you tell me of a home in Bath, Bristol, or the neighbourhood where an elderly widow could be received who is crippled in her lower limbs only? She has sons who would pay towards her support.—*S. M. C. B.*

Possibly Miss Jeffes, who is connected with the Girls' Friendly Society, Russel House, Russel Street, Bath, would advise you as regards that neighbourhood; and the Lady Superintendent, the Orthopædic Hospital and Home for Crippled Children, Grove Road, Redland, for Bristol. The Little Sisters of the Poor have a home at Cotham Park, Bristol.

*Holt-Ockley Association.*

(172) Can you please give me the address of the Holt-Ockley Association?—*G. W.*

Write to the Lady Superintendent, Miss Bullock, Nurses' Hostel, Station Road, Horsham.

*Instruction in Massage.*

(173) Will you please tell me in what paper I shall find the address of a private instructor in massage in Manchester?—*A. L.*

Write to the Incorporated Society of Trained Masseuses, 12 Buckingham Street, Strand, London, W.C., for advice.

*Registration.*

(174) I was trained fourteen years ago in the Royal Glasgow Maternity Hospital, and have since been employed by the Royal Scottish Nursing Institute. Do I need to be registered?

Write to the Secretary, Central Midwives Board, Suffolk Street, Pall Mall, London, S.W.

*Post of Nurse-Companion.*

(175) Five years ago I had two years' training at St. Thomas's Hospital, and left through ill health. I have always kept up my interest in the nursing world. Should I be justified in taking a situation as nurse-companion?—*M. J. F.*

Yes, if you state the circumstances.

*Training in Midwifery.*

(176) Can I get free training in midwifery in a hospital for soldiers' wives?—*S. W.*

We know of no hospital where a free training in midwifery can be obtained.

### Handbooks for Nurses.

"How to Become a Nurse: The Nursing Profession." 2s. net; 2s. 4d. post free.

"The Nurses' Dictionary." By Honnor Morten. 2s. post free.

"A Handbook for Nurses." By J. K. Watson, M.D. 5s. net; 5s. 4d. post free.

"A Practical Guide to Surgical Bandaging and Dressings." By W. Johnson Smith, F.R.C.S. 2s. post free.

"The Art of Massage." By A. Creighton Hale. 6s. post free.

"Preparation for Operation in Private Houses." By Stanmore Bishop, F.R.C.S. 6d. post free.

The above works are obtainable of all booksellers, or direct from The Scientific Press, Limited, 28 & 29 Southampton Street, Strand, London, W.C.

### For Reading to the Sick.

"TEN CLEANSED, AND ONLY ONE REMAIN!"

TEN, and only one remain!
Who would have thought our nature's stain
Was dyed so foul, so deep in grain.
Even He who reads the heart—

Knows what He gave and what we lost,
Sin's forfeit, and redemption's cost—
By a short pang of wonder crossed
Seems at the sight to start:

How should we gaze in trance of fear!
Yet shines the light as thrilling clear
From Heaven upon that scroll severe,
"Ten cleansed and one remain!"

No surer would the blessing prove
Of humbled hearts, that own Thy love,
Should choral welcome from above
Visit our senses plain:

Than by Thy placid voice and brow,
With healing first, with comfort now,
Turned upon him, who hastes to bow
Before Thee, heart and knee;
"Oh! thou, who only wouldst be blest,
"On thee alone My blessing rest!"

*Keble.*

When we read this Gospel we are ready, perhaps, to wish we were like these ten poor lepers. We lie and think of our aches and our pains, and wish the Lord would pass by and cure us, as He cured these lepers of the most dreadful disease in the world.

We do not see Him pass, but He *does* come to us still. He looks on us with eyes full of pity; often He blesses the doctor's skill and the nurse's skill, so that by and by, though not all in a minute, we are "cleansed," and we go home cured. And what then, dear friends? Do we go back to the work and the worries, and forget all about our Lord, and about the doctor and the nurse whom He taught to cure us? Or do we glorify God, and fall down before Jesus and give Him thanks? Ah, for one of us that does that, are there not nine of us who forget to thank Him at all?

A great surgeon once saved the life of a poor woman, not by a word, like our Lord, but by a skilful operation. She was thankful, and wrote every Christmas to tell him she was happy in the life he had preserved for her. But in time she grew tired of writing. She said it was no use to worry the great man with so many thanks. Time passed, and she feared her illness was returning, and went again to consult the surgeon. He told her she was mistaken, she was cured, and he was glad. "But," he added, "I had no letter last Christmas, and I missed it; yes, I did miss it." His patient's heart was filled with sorrow. "You shall never miss it again," she cried.—*B. Waugh.*

A thankful spirit towards God will induce a generous spirit towards man. One very true element of thankfulness is to desire that "His way may be known upon earth"; and your thanksgiving will be all the truer and more fervent if in it you commend, not only Christ's Church, but all those who have not yet known Him, to His Redeeming Love.

Every action through the day might be moulded by this spirit of thanksgiving; and nothing surely would tend more to keep up a consciousness of that blessed Presence, than to find it influencing the will, the affections, and the outward deeds.

